# INFLUENCE OF CHANGES IN MEMORY AND THINKING ON PREFERENCES AND SATISFACTION OF HEALTH CARE COMMUNICATIONS

**DOI:** 10.1093/geroni/igad104.2948

**Published:** 2023-12-21

**Authors:** Andrea Sillner, Marie Boltz, Diane Berish, Nikki Hill

**Affiliations:** Pennsylvania State University, State College, Pennsylvania, United States; Pennsylvania State University, State College, Pennsylvania, United States; The Pennsylvania State University, University Park, Pennsylvania, United States; The Pennsylvania State University, University Park, Pennsylvania, United States

## Abstract

Communication about changes in symptoms or healthcare management strategies is common between older adults and their healthcare providers, such as physicians and registered nurses. Healthcare communication can occur in the context of an in-person visit or using technology through telehealth or a healthcare portal. Understanding factors that influence preferences for healthcare communication and satisfaction are important for patients and providers. For example, changes in memory and thinking may impact older adults’ ability to communicate healthcare needs or to understand what is being told to them about their health conditions. The aim of this study was to understand the influence of cognitive changes on preferences and satisfaction with communications in a sample of adults over the age of 50, living in the community, who reported on changes in memory and thinking over the last 5 years (N=289). Results showed that older adults who reported a decline in their memory and thinking over the last 5 years were significantly more likely to report that their healthcare communication needs were not met. They were also significantly less likely to be satisfied with their healthcare communications. These important findings demonstrate the need to further explore relationships between self-reported cognitive changes and communication with healthcare providers. This can inform psycho-social intervention development, ultimately enhancing healthcare communication to meet older patient preferences and improve satisfaction with the overall goal of impacting healthcare outcomes.

